# Development and validation of a deep neural network model to predict postoperative mortality, acute kidney injury, and reintubation using a single feature set

**DOI:** 10.1038/s41746-020-0248-0

**Published:** 2020-04-20

**Authors:** Ira S. Hofer, Christine Lee, Eilon Gabel, Pierre Baldi, Maxime Cannesson

**Affiliations:** 1 Department of Anesthesiology and Perioperative Medicine, David Geffen School of Medicine at UCLA, Los Angeles, CA USA; 2 Department of Biomedical Engineering, University of California Irvine, Irvine, CA USA; 3 Department of Computer Sciences, University of California Irvine, Irvine, CA USA

**Keywords:** Disease-free survival, Health policy, Translational research

## Abstract

During the perioperative period patients often suffer complications, including acute kidney injury (AKI), reintubation, and mortality. In order to effectively prevent these complications, high-risk patients must be readily identified. However, most current risk scores are designed to predict a single postoperative complication and often lack specificity on the patient level. In other fields, machine learning (ML) has been shown to successfully create models to predict multiple end points using a single input feature set. We hypothesized that ML can be used to create models to predict postoperative mortality, AKI, reintubation, and a combined outcome using a single set of features available at the end of surgery. A set of 46 features available at the end of surgery, including drug dosing, blood loss, vital signs, and others were extracted. Additionally, six additional features accounting for total intraoperative hypotension were extracted and trialed for different models. A total of 59,981 surgical procedures met inclusion criteria and the deep neural networks (DNN) were trained on 80% of the data, with 20% reserved for testing. The network performances were then compared to ASA Physical Status. In addition to creating separate models for each outcome, a multitask learning model was trialed that used information on all outcomes to predict the likelihood of each outcome individually. The overall rate of the examined complications in this data set was 0.79% for mortality, 22.3% (of 21,676 patients with creatinine values) for AKI, and 1.1% for reintubation. Overall, there was significant overlap between the various model types for each outcome, with no one modeling technique consistently performing the best. However, the best DNN models did beat the ASA score for all outcomes other than mortality. The highest area under the receiver operating characteristic curve (AUC) models were 0.792 (0.775–0.808) for AKI, 0.879 (0.851–0.905) for reintubation, 0.907 (0.872–0.938) for mortality, and 0.874 (0.864–0.866) for any outcome. The ASA score alone achieved AUCs of 0.652 (0.636–0.669) for AKI, 0.787 (0.757–0.818) for reintubation, 0.839 (0.804–0.875) for mortality, and 0.76 (0.748–0.773) for any outcome. Overall, the DNN architecture was able to create models that outperformed the ASA physical status to predict all outcomes based on a single feature set, consisting of objective data available at the end of surgery. No one model architecture consistently performed the best.

## Introduction

The perioperative period contains significant risk, where clinical instability is the norm more than the exception^1,2^. Up to 43% of surgical patients may exhibit some kind of perioperative complication^3–16^, and short-term morbidities are associated with longer-term outcomes. A recently published manuscript has demonstrated that perioperative mortality is the third leading cause of death internationally^17^.

Although perioperative care can help prevent these complications^4,18^, clinicians often struggle to identify those patients at highest risk of complications without performing time-consuming chart reviews^19^. This has led to the adoption of risk scoring systems^20,21^; however, most current risk scores are focused on individual complications^22,23^, and tend to use simplistic point systems to allow for easy application^21,22^. Recently, machine learning (ML) has shown promise as a way to integrate large amounts of data in an automated fashion, in order to predict the risk of perioperative outcomes^24–26^.

Advantages of ML include the ability of a single set of inputs (features) to simultaneously used to predict multiple end points, and the ability to automate these models and integrate results directly into electronic health records (EHRs). While the early results of studies using ML techniques on EHR data to predict outcomes are promising, creating scalable progress in the field requires a better understanding of which techniques are most likely to be successful. One particular technique of interest is multitask learning, where the models can use information on one outcome to help improve the prediction of an associated outcome—for example using data on acute kidney injury (AKI) prediction to help predict mortality. This is of particular interest in the perioperative period because clinicians and patients are not interested in the risk of a singular event, but rather a constellation of key outcomes (i.e., mortality, kidney injury, respiratory dysfunction, etc.).

In this manuscript, we hypothesize that a deep neural network (DNN) can be used to create a model that predicts multiple postoperative outcomes—specifically AKI, reintubation, in-hospital mortality, and the composite outcome of any postoperative event—based on a single feature set containing data that can be easily extracted from an electronic medical record (EMR) at the end of surgery. We first report the results of models that predict each of the outcomes individually, and then report the results of a combined model that uses multitask learning to create a single model to predict all three outcomes. Lastly, we slightly alter our feature set to add some features known to be highly associated with the outcomes of interest to see if this improves model performance. As a primary outcome measure, we compare these models to each other and to the ASA physical status score, logistic regression (LR), and the Risk Stratification Index (RSI), and the Risk Quantification Index (RQI)^27^ based on the area under the receiver operating characteristic curve (AUC). As secondary outcomes, we look at the F1 score, sensitivity, specificity, and precision of the models.

## Results

### Patient characteristics

During the study period, 59,981 cases met inclusion criteria. A total of 38,305 of these patients lacked either preoperative or postoperative serum creatinine (Cr_S_), and thus AKI class could not be determined. The overall rates of the examined complications in this data set was 0.79% for mortality, 22.3% (of 21,676 patients with Cr values) for AKI, and 1.1% for reintubation. Detailed patient characteristics (including the rates of AKI, reintubation, and mortality) are shown in Table 1.

**Table 1:** Description of demographic features.

	Train	Test
# Patients	47,985	11,996
Age	56 ± 17	56 ± 94
EBL	96 ± 539	18 ± 410
# With Aline	8583	2135
# With PA	1641	430
# With CVC	2443	635
ASA score		
1	3022	762
2	17,930	4477
3	23,960	5985
4	2910	735
5	144	30
6	4	0
Unknown	15	7
Primary CPT by specialty		
Gastroenterology	6615 (13.8%)	1614 (13.5%)
General Surgery	6552 (13.7%)	1646 (13.7%)
Urology	4005 (8.3%)	1062 (8.9%)
Orthopedics	3916 (8.2%)	979 (8.2%)
Neurosurgery	3686 (7.7%)	916 (7.6%)
Otolaryngology	3268 (6.8%)	860 (7.2%)
Obstetrics and Gynecology	2630 (5.5%)	672 (5.6%)
Vascular Surgery	1834 (3.8%)	445 (3.7%)
Cardiac Surgery	1396 (2.9%)	372 (3.1%)
Thoracic Surgery	1095 (2.3%)	273 (2.3%)
Other	8497 (17.7%)	2049 (17.1%)
Unknown	4491 (9.4%)	1108 (9.2%)
AKI		
Class 1	2501 (5.21%)	622 (5.19%)
Class 2	369 (0.77%)	99 (0.83%)
Class 3	1001 (2.09%)	246 (2.05%)
Null	30616 (63.8%)	7689 (64.1%)
Reintubation	548 (1.14%)	159 (1.33%)
Mortality	389 (0.81%)	87 (0.73%)

### Individual model performance

**Fig. 1:**
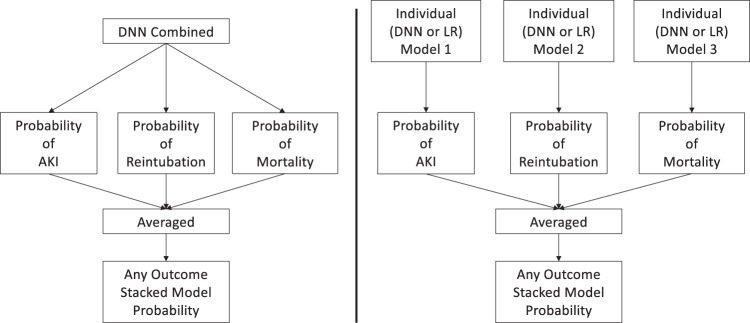
Visual depiction of the any outcome stacked models. Summary figure describing the stacked “any” postoperative outcome models for the combined deep neural networks (DNN combined) trained to output probabilities of all three outcomes vs the deep neural networks (DNN individual) and logistic regression (LR) models that were individually trained per outcome.

As a baseline, models were created to predict each outcome separately (i.e., AKI, mortality, reintubation, or any outcome) using a DNN original feature set (DNN OFS). The models all performed well with AUCs of 0.780 (95% CI 0.763–0.796) for AKI, 0.879 (95% CI 0.851–0.905) for reintubation, 0.895 (95% CI .854–0.930) for mortality, and 0.866 (95% CI 0.855–0.878) for any outcome. Of note, the AKI models had smaller training and validation datasets due to the missing Cr values for some patients. These results as well those for the other models can be found in Table 2. Figure 1 shows the ROC plots for the various models for every outcome.

**Table 2:** AUC for prediction of acute kidney injury (AKI), reintubation, mortality, and any outcome with 95% CIs for the test set (*N* = 11,996) for the ASA score, logistic regression (LR) models, deep neural networks predicting individual outcomes (DNN individual), and deep neural networks predicting all three outcomes (DNN combined).

Score	AKI^a^	Reintubation	Mortality	Any outcome
ASA	0.652 (0.636–0.669)	0.787 (0.757–0.818)	0.839 (0.804–0.875)	0.76 (0.748–0.773)
RQI^b^	0.652 (0.623–0.683)	0.878 (0.842–0.909)	0.907 (0.86–0.942)	0.8 (0.778–0.821)
RSI^c^	0.594 (0.571–0.615)	0.829 (0.783–0.873)	0.97 (0.944–0.99)	0.597 (0.576–0.621)

Model type	AKI^a^	Reintubation	Mortality	Any outcome (stacked model)
LR OFS	0.767 (0.748–0.785)	0.856 (0.82–0.888)	0.9 (0.865–0.93)	0.843 (0.829–0.857)
LR OFS + MAP	0.767 (0.749–0.785)	0.855 (0.818–0.887)	0.898 (0.863–0.93)	0.843 (0.829–0.857)
LR RFS	0.767 (0.748–0.785)	0.862 (0.827–0.894)	0.899 (0.864–0.93)	0.843 (0.829–0.858)
DNN Individual OFS	0.78 (0.763–0.796)	**0.879 (0.851**–**0.905)**	0.895 (0.854–0.93)	0.866 (0.855–0.878)
DNN Individual OFS + MAP	**0.792 (0.775**–**0.808)**	0.876 (0.848–0.902)	0.903 (0.871–0.933)	**0.874 (0.864**–**0.886)**
DNN Individual RFS	0.783 (0.766–0.799)	**0.879 (0.851**–**0.905)**	0.9 (0.865–0.931)	0.866 (0.854–0.878)
DNN Combined OFS	0.785 (0.767–0.801)	0.858 (0.829–0.886)	**0.907 (0.872**–**0.938)**	0.865 (0.854–0.877)
DNN Combined OFS + MAP	0.783 (0.765–0.8)	0.84 (0.808–0.872)	0.906 (0.87–0.937)	0.86 (0.848–0.872)
DNN Combined RFS	0.789 (0.772–0.806)	0.842 (0.811–0.871)	0.906 (0.87–0.937)	0.852 (0.84–0.864)

Each model was also evaluated for each feature set combination of original feature set (OFS), OFS + the minimum MAP features (OFS + MAP), and reduced feature set (RFS). Note that for the LR and individual models, there is one model per outcome and the predicted outcome probabilities from each model is stacked to predict any outcome. For the combined models, there is one model for all three outcomes and those probabilities are stacked to predict any outcome. Bold results indicate the best AUC for that measure.

^a^It should be noted that AKI labels were only available for 4307 of the test patients, and so all AUCs reflect results for only those patients with AKI labels.

^b^RQI was calculated on 5591 test patients (63 reintubation, 38 mortality, and 491 any label); and on 2319 test patients with AKI labels (445 positive).

^c^RSI was calculated on 11,939 test patients (159 reintubation, 86 mortality, and 1066 any label); and on 4294 test patients with AKI labels (967 positive).

### Combined model and changes in model features

In an effort to improve model performance, we attempted to train a combined model that would output the risk of each individual outcome. The thought was that in using a model that had information on all of the outcomes the model could “learn” from one outcome, in order to predict the others. In fact, the AUCs of these models were not better than those for the individual outcomes: 0.785 (95% CI 0.767–0.801) for AKI, 0.858 (95% CI 0.829–0.886) for reintubation, 0.907 (95% CI 0.872–0.938) for mortality, and 0.865 (95% CI 0.854–0.877) for any outcome.

In another effort to improve the model performance, we examined the effect of two changes in input features. In the first change, given the literature on associations between intraoperative hypotension and outcomes, we added data on the duration of intraoperative hypotension. In the case of the individual DNN models, these additions did not improve the model. For the combined models, the addition of the mean arterial pressure (MAP) data actually trended toward reducing the AUCs in some instances. In the second modification, we reduced the feature set to remove those features with a Pearson correlation coefficient > 0.9. This feature reduction did not change the results of the model for either the individual or combined models. All these results are contained in Table 2 and Fig. 1.

### Comparison to the ASA score, LR, RSI, and RQI

For the AKI and any outcome end points all DNN models outperformed the ASA score, RSI, and RQI (best AKI model 0.792 (0.775–0.808) vs 0.652 (0.636–0.669) for ASA, 0.652 (0.623–0.683) for RQI and 0.594 (0.571–0.615) for RSI, and any outcome 0.874 (0.864–0.886) vs 0.76 (0.748–0.773) for ASA). For reintubation most, but not all, models outperformed the ASA score (best model 0.879 (0.851–0.905) vs 0.787 (0.757–0.818) for ASA, but did not outperform the RSI and RQI 0.878 (0.842–0.909) for RQI and 0.829 (0.783–0.873) for RSI. In the case of mortality, no model outperformed the ASA score or RQI (0.907 (0.872–0.938) for best model vs 0.839 (0.804–0.875) for ASA score for RQI 0.8 (0.778–0.821), but all models outperformed the RSI 0.597 (0.576–0.621). In comparison with LR, the DNN models performed similarly to LR.

### Choosing a threshold

For a given model, the threshold can be adjusted so as to optimize different parameters, i.e., a more sensitive model vs a more specific model. In Table 3, we report the threshold, sensitivity, specificity, precision, and other relevant data for each model, where the threshold is chosen to optimize the *F*1 score (which is a balance of precision and recall). Results for optimizing for other end points are shown in Supplementary Table 3a–c. The thresholds for the *F*1 scores varied considerably between the different model types, as well as across outcomes. For example, thresholds for the mortality model ranged from 0.55 to 0.975 (or 5 for the ASA model). Depending on the end point, the various threshold and model combinations led to significant variations in the best *F*1 scores.

**Table 3:** Best threshold chosen by highest *F*1 score.

AKI^a^										
Score	Threshold	*F*1 score (95% CI)	Sensitivity (95% CI)	Specificity (95% CI)	Precision (95% CI)	TN	FP	FN	TP	Accuracy (%)
ASA	3	0.412 (0.393–0.43)	0.914 (0.896–0.93)	0.27 (0.255–0.284)	0.266 (0.251–0.281)	901	2439	83	884	41.4
**LR OFS**	**0.273071**	**0.538 (0.512**–**0.563)**	0.631 (0.597–0.661)	0.793 (0.78–0.807)	0.469 (0.442–0.497)	2650	690	357	610	75.7
LR OFS + MAP features	0.27574	0.537 (0.512–0.563)	0.624 (0.59–0.654)	0.798 (0.785–0.812)	0.472 (0.444–0.5)	2666	674	364	603	75.9
LR RFS	0.287606	0.537 (0.51–0.563)	0.607 (0.575–0.637)	0.811 (0.798–0.823)	0.482 (0.454–0.511)	2708	632	380	587	76.5
DNN individual OFS	0.408436	0.545 (0.52–0.569)	0.654 (0.622–0.682)	0.784 (0.77–0.798)	0.467 (0.441–0.493)	2618	722	335	632	75.5
**DNN individual OFS** **+** **MAP features**	**0.481765**	**0.559 (0.533**–**0.587)**	0.548 (0.515–0.579)	0.881 (0.87–0.892)	0.571 (0.542–0.603)	2942	398	437	530	80.6
DNN individual RFS	0.406397	0.542 (0.516–0.568)	0.618 (0.586–0.648)	0.808 (0.794–0.821)	0.483 (0.455–0.51)	2699	641	369	598	76.5
DNN combined OFS	0.906036	0.548 (0.521–0.575)	0.568 (0.536–0.598)	0.854 (0.843–0.865)	0.53 (0.501–0.559)	2853	487	418	549	79.0
DNN combined OFS + MAP features	0.901522	0.549 (0.524–0.575)	0.58 (0.55–0.61)	0.846 (0.833–0.857)	0.521 (0.493–0.552)	2825	515	406	561	78.6
DNN combined RFS	0.869984	0.557 (0.53–0.583)	0.575 (0.543–0.606)	0.858 (0.846–0.87)	0.539 (0.51–0.569)	2865	475	411	556	79.4

Reintubation										
Score	Threshold	*F*1 score (95% CI)	Sensitivity (95% CI)	Specificity (95% CI)	Precision (95% CI)	TN	FP	FN	TP	Accuracy (%)
ASA	4	0.152 (0.121–0.182)	0.44 (0.361–0.517)	0.941 (0.937–0.945)	0.092 (0.072–0.112)	11,142	695	89	70	93.5
LR OFS	0.08	0.21 (0.157–0.261)	0.296 (0.223–0.366)	0.98 (0.977–0.982)	0.163 (0.121–0.207)	11,595	242	112	47	97.0
**LR OFS** **+** **MAP features**	**0.081**	**0.223 (0.168**–**0.276)**	0.314 (0.24–0.389)	0.98 (0.977–0.982)	0.172 (0.129–0.22)	11,597	240	109	50	97.1
LR RFS	0.079193	0.211 (0.161–0.262)	0.302 (0.231–0.375)	0.979 (0.977–0.982)	0.163 (0.121–0.207)	11,590	247	111	48	97.0
DNN individual OFS	0.715748	0.21 (0.16–0.257)	0.333 (0.257–0.406)	0.975 (0.972–0.978)	0.153 (0.115–0.192)	11,544	293	106	53	96.7
DNN individual OFS + MAP features	0.734977	0.197 (0.149–0.243)	0.321 (0.247–0.397)	0.974 (0.971–0.977)	0.142 (0.104–0.179)	11,530	307	108	51	96.5
DNN individual RFS	0.687943	0.22 (0.17–0.269)	0.371 (0.297–0.445)	0.973 (0.97–0.976)	0.156 (0.117–0.196)	11,518	319	100	59	96.5
DNN combined OFS	0.769994	0.206 (0.164–0.252)	0.352 (0.284–0.428)	0.972 (0.969–0.975)	0.145 (0.113–0.181)	11,508	329	103	56	96.4
**DNN combined OFS** **+** **MAP features**	**0.784518**	**0.228 (0.179**–**0.278)**	0.34 (0.271–0.414)	0.978 (0.975–0.981)	0.171 (0.131–0.215)	11,576	261	105	54	96.9
DNN combined RFS	0.746933	0.213 (0.166–0.263)	0.289 (0.221–0.36)	0.981 (0.978–0.983)	0.168 (0.128–0.214)	11,610	227	113	46	97.2

Mortality										
Score	Threshold	*F*1 score (95% CI)	Sensitivity (95% CI)	Specificity (95% CI)	Precision (95% CI)	TN	FP	FN	TP	Accuracy (%)
ASA	5	0.239 (0.138–0.356)	0.161 (0.088–0.253)	0.999 (0.998–0.999)	0.467 (0.3–0.667)	11,893	16	73	14	99.3
LR OFS	0.194	0.306 (0.208–0.402)	0.253 (0.167–0.346)	0.997 (0.996–0.998)	0.386 (0.265–0.516)	11,874	35	65	22	99.2
**LR OFS** **+ MAP features**	**0.203**	**0.306 (0.212**–**0.4)**	0.253 (0.17–0.345)	0.997 (0.996–0.998)	0.386 (0.267–0.519)	11,874	35	65	22	99.2
LR RFS	0.135	0.287 (0.196–0.375)	0.299 (0.202–0.404)	0.994 (0.993–0.996)	0.277 (0.187–0.372)	11,841	68	61	26	98.9
**DNN individual OFS**	**0.59**	**0.294 (0.202**–**0.389)**	0.276 (0.188–0.383)	0.996 (0.994–0.997)	0.316 (0.215–0.429)	11,857	52	63	24	99.0
DNN individual OFS + MAP features	0.587	0.268 (0.181–0.36)	0.253 (0.167–0.356)	0.995 (0.994–0.996)	0.286 (0.192–0.391)	11,854	55	65	22	99.0
DNN individual RFS	0.55	0.278 (0.204–0.357)	0.368 (0.276–0.474)	0.991 (0.989–0.992)	0.224 (0.16–0.291)	11,798	111	55	32	98.6
DNN combined OFS	0.950117	0.271 (0.175–0.367)	0.218 (0.136–0.312)	0.997 (0.996–0.998)	0.358 (0.231–0.482)	11,875	34	68	19	99.1
DNN combined OFS + MAP features	0.975254	0.239 (0.138–0.344)	0.161 (0.089–0.244)	0.999 (0.998–0.999)	0.467 (0.294–0.64)	11,893	16	73	14	99.3
DNN combined RFS	0.868749	0.267 (0.183–0.346)	0.299 (0.205–0.393)	0.993 (0.992–0.995)	0.241 (0.164–0.325)	11,827	82	61	26	98.8

Any outcome										
Score	Threshold	*F*1 score (95% CI)	Sensitivity (95% CI)	Specificity (95% CI)	Precision (95% CI)	TN	FP	FN	TP	Accuracy (%)
ASA	4	0.36 (0.335–0.387)	0.309 (0.283–0.337)	0.96 (0.957–0.964)	0.431 (0.399–0.468)	10,494	435	737	330	90.2
**LR OFS**	**0.122592**	**0.504 (0.48**–**0.529)**	0.542 (0.513–0.572)	0.941 (0.936–0.945)	0.471 (0.445–0.498)	10,280	649	489	578	90.5
LR OFS + MAP features	0.12059	0.503 (0.48–0.53)	0.549 (0.521–0.58)	0.938 (0.934–0.943)	0.465 (0.439–0.492)	10,254	675	481	586	90.4
LR RFS	0.124499	0.503 (0.479–0.529)	0.532 (0.505–0.563)	0.943 (0.939–0.947)	0.477 (0.449–0.504)	10,305	624	499	568	90.6
DNN individual OFS	0.411454	0.479 (0.455–0.504)	0.515 (0.487–0.545)	0.938 (0.934–0.942)	0.448 (0.422–0.475)	10,252	677	518	549	90.0
**DNN individual OFS** **+** **MAP features**	**0.395795**	**0.482 (0.46**–**0.506)**	0.584 (0.555–0.616)	0.918 (0.913–0.923)	0.41 (0.386–0.434)	10,033	896	444	623	88.8
DNN individual RFS	0.402621	0.473 (0.449–0.498)	0.535 (0.508–0.567)	0.929 (0.924–0.934)	0.424 (0.399–0.452)	10,153	776	496	571	89.4
DNN combined OFS	0.710049	0.47 (0.445–0.496)	0.503 (0.475–0.534)	0.938 (0.934–0.942)	0.441 (0.412–0.47)	10,249	680	530	537	89.9
DNN combined OFS + MAP features	0.678431	0.475 (0.452–0.5)	0.587 (0.558–0.616)	0.914 (0.909–0.919)	0.399 (0.376–0.424)	9988	941	441	626	88.5
DNN combined RFS	0.632316	0.446 (0.423–0.469)	0.565 (0.535–0.595)	0.905 (0.9–0.911)	0.368 (0.345–0.39)	9894	1035	464	603	87.5

Comparison of *F*1 score, sensitivity, and specificity with best thresholds for acute kidney injury (AKI), reintubation, mortality, and any outcome with 95% CIs for the test set (*N* = 11,996) for the ASA score, logistic regression (LR) models, deep neural networks predicting individual outcomes (DNN individual), and deep neural networks predicting all three outcomes (DNN combined). Each model was also evaluated for each feature set combination of original feature set (OFS), OFS + the minimum MAP features (OFS + MAP), and reduced feature set (RFS). Note that for the LR and individual models, there is one model per outcome and the predicted outcome probabilities from each model is stacked to predict any outcome. For the combined models, there is one model for all three outcomes and those probabilities are stacked to predict any outcome.

^a^It should be noted that AKI labels were only available for 4307 of the test patients, and so all results for AKI are from those patients with AKI labels. Bolded are the best *F*1 scores for logistic regression and DNN models.

### Precision, recall, and specificity

Table 3 demonstrates the precision, sensitivity, specificity, and other relevant statistics for each model, where a threshold was chosen to optimize the *F*1 score, and Fig. 1 demonstrates the precision-recall curve for the various models. Overall, while the AUCs of the various models were remarkably similar, at different thresholds there was significant variation in measures like sensitivity, specificity, and precision between the various outcomes, and at times between models for a single outcome. For example, sensitivity for the individual DNN OFS model ranged from 0.654 (95% CI 0.622–0.682) for the AKI model to 0.276 (95% CI 0.188–0.383) for the mortality model, while precision results ranged from 0.266–0.539 for the AKI model. Overall, the area under the precision-recall curve was in the 0.5 range for the AKI and any label models, and much lower for the mortality and reintubation models. Supplementary Table 3a–c shows the relevant statistics for thresholds chosen to optimize sensitivity, specificity closest to 0.9, and precision.

### Comparison of model accuracy using the McNemar test

In order to asses the ability of the individual DNN models as compared to LR models, and the individual DNN models to the combined DNN models, we used the McNemar test to look at overall model accuracy. All results were based on the threshold that optimized the *F*1 score for that model. These results are shown in Table 4a, b. In general there was no clear trend of superior accuracy between the combined models and either the LR or individual models. If we compare the LR with the original features to the best performing DNN models, we see that there was a significant difference for AKI, mortality, and any outcome but not for reintubation. Of these the DNN model preformed better for both mortality and any outcome but not AKI. In comparing the individual vs combined models, the individual models tended to have better accuracy for AKI, while the combined models tended to have better accuracy for the other outcomes.

**Table 4:** **a** McNemar test results comparing logistic regression (LR) models and deep neural network (DNN) models classification errors when choosing best thresholds by the highest *F*1 score. **b** McNemar test results comparing individual DNN to combined DNN.

		AKI^a^		Reintubation		Mortality		Any outcome	
Logistic regression model	DNN model	*p*	*p* < 0.05	*p*	*p* < 0.05	*p*	*p* < 0.05	*p*	*p* < 0.05
LR OFS	DNN combined RFS	4.62E−15	TRUE	4.39E−01	FALSE	1.77E−06	TRUE	5.92E−34	TRUE
LR OFS	DNN combined OFS	1.34E−11	TRUE	8.42E−06	TRUE	8.78E−01	FALSE	6.05E−03	TRUE
LR OFS	DNN combined OFS + MAP features	8.01E−10	TRUE	5.08E−01	FALSE	1.26E−01	FALSE	2.54E−21	TRUE
LR OFS	DNN individual OFS	5.92E−01	FALSE	5.72E−04	TRUE	2.01E−02	TRUE	1.90E−02	TRUE
LR OFS	DNN individual RFS	3.34E−02	TRUE	1.33E−06	TRUE	2.12E−12	TRUE	1.32E−07	TRUE
LR OFS	DNN individual OFS + MAP Features	**3.38E**−**22**	**TRUE**	5.29E−06	TRUE	2.89E−03	TRUE	**7.37E**−**16**	**TRUE**
LR RFS	DNN combined RFS	2.39E−10	TRUE	3.15E−01	FALSE	1.75E−01	FALSE	7.52E−04	TRUE
LR RFS	DNN combined OFS	7.48E−08	TRUE	3.12E−05	TRUE	1.82E−03	TRUE	4.49E−24	TRUE
LR RFS	DNN combined OFS + MAP features	3.63E−06	TRUE	6.80E−01	FALSE	8.58E−06	TRUE	3.67E−37	TRUE
LR RFS	DNN individual OFS	1.28E−02	TRUE	1.76E−03	TRUE	8.14E−02	FALSE	2.86E−03	TRUE
LR RFS	DNN individual RFS	9.53E−01	FALSE	3.56E−06	TRUE	4.77E−05	TRUE	3.25E−09	TRUE
LR RFS	DNN individual OFS + MAP features	1.36E−17	TRUE	3.03E−05	TRUE	3.21E−01	FALSE	6.21E−18	TRUE
LR OFS + MAP features	DNN combined RFS	4.54E−14	TRUE	6.38E−01	FALSE	1.77E−06	TRUE	4.11E−02	TRUE
LR OFS + MAP features	DNN combined OFS	7.89E−11	TRUE	2.51E−06	TRUE	8.83E−01	FALSE	1.49E−18	TRUE
LR OFS + MAP features	DNN combined OFS + MAP features	7.09E−09	TRUE	**3.43E**−**01**	**FALSE**	1.35E−01	FALSE	2.81E−31	TRUE
LR OFS + MAP features	DNN individual OFS	2.90E−01	FALSE	1.41E−04	TRUE	**3.57E**−**02**	**TRUE**	1.15E−01	FALSE
LR OFS + MAP features	DNN individual RFS	1.09E−01	FALSE	3.59E−07	TRUE	4.03E−12	TRUE	5.36E−06	TRUE
LR OFS + MAP features	DNN individual OFS + MAP features	3.81E−21	TRUE	9.69E−07	TRUE	6.60E−03	TRUE	2.09E−13	TRUE

		AKI^a^		Reintubation		Mortality		Any Outcome	
DNN individual	DNN combined	*p*	*p* < 0.05	*p*	*p* < 0.05	*p*	*p* < 0.05	*p*	*p* < 0.05
DNN individual OFS	DNN combined OFS	7.78E−03	TRUE	1.00E+00	FALSE	6.16E−01	FALSE	5.58E−01	FALSE
DNN individual OFS	DNN combined OFS + MAP features	2.50E−01	FALSE	6.54E−40	TRUE	1.67E−38	TRUE	7.99E−13	TRUE
DNN individual OFS	DNN combined RFS	1.34E−01	FALSE	2.74E−51	TRUE	2.46E−47	TRUE	9.38E−28	TRUE
DNN individual RFS	DNN combined OFS	1.42E−07	TRUE	2.76E−05	TRUE	1.05E−07	TRUE	1.50E−02	TRUE
DNN individual RFS	DNN combined OFS + MAP features	1.42E−01	FALSE	1.93E−18	TRUE	2.36E−15	TRUE	4.71E−05	TRUE
DNN individual RFS	DNN combined RFS	2.54E−01	FALSE	3.36E−29	TRUE	1.21E−23	TRUE	2.92E−16	TRUE
DNN individual OFS + MAP features	DNN combined OFS	1.80E−10	TRUE	1.97E−27	TRUE	4.81E−31	TRUE	4.93E−07	TRUE
DNN individual OFS + MAP features	DNN combined OFS + MAP features	2.51E−03	TRUE	1.28E−02	TRUE	4.41E−02	TRUE	1.06E−01	FALSE
DNN individual OFS + MAP features	DNN combined RFS	1.04E−02	TRUE	4.93E−07	TRUE	2.40E−05	TRUE	8.26E−09	TRUE

McNemar test *p* values < 0.05 were considered significant, indicating that the classifiers have significantly different proportion of errors when classifying acute kidney injury (AKI), reintubation, mortality, or any outcome for the test set (*N* = 11,996) when comparing the logistic regression (LR) models, deep neural networks predicting individual outcomes (DNN individual), and deep neural networks predicting all three outcomes (DNN combined). Each model was also evaluated for each feature set combination of original feature set (OFS), OFS + the minimum MAP features (OFS + MAP), and reduced feature set (RFS). Note that for the LR and individual models, there is one model per outcome and the predicted outcome probabilities from each model is stacked to predict any outcome. For the combined models, there is one model for all three outcomes and those probabilities are stacked to predict any outcome.

Bolded results are the smallest *p* values for the given outcome.

An example of how to interpret this table is: for correctly classifying any outcome, all LR and DNN models were significantly different (*p* < 0.05) from each other except for LR OFS + MAP and DNN Individual OFS. The best performing *F*1 score LR model was LR OFS (*F*1 score 0.504, sensitivity 0.542, specificity 0.941, and precision 0.471) and the best performing DNN model was DNN individual OFS + MAP (*F*1 score 0.482; sensitivity 0.584; specificity 0.918; and precision 0.41).

^a^It should be noted that AKI labels were only available for 4307 of the test patients, and so all results for AKI are from those patients with AKI labels.

### Correlation between results

**Fig. 2:**
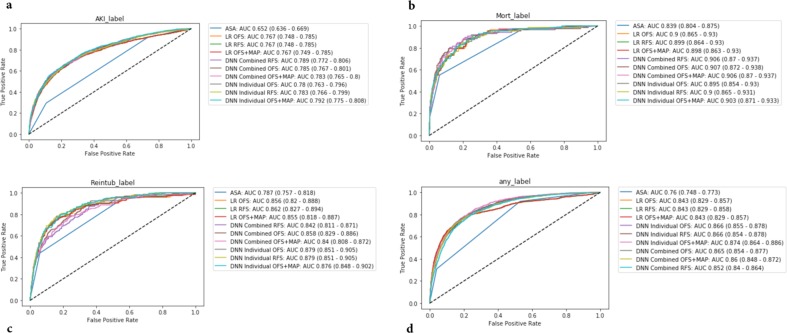
ROC Curves for AKI, mortality, reintubation and any outcome. ROC Curves for AKI (**a**), mortality (**b**), reintubation (**c**) and any outcome (**d**). Receiver operator characteristic curves for acute kidney injury (AKI), reintubation, mortality, and any outcome for the test set (*N* = 11,996) for the ASA score, logistic regression (LR) models, deep neural networks predicting individual outcomes (DNN individual), and deep neural networks predicting all three outcomes (DNN combined). Each model was also evaluated for each feature set combination of original feature set (OFS), OFS + the minimum MAP features (OFS + MAP), and reduced feature set (RFS). Note that for the LR and individual models, there is one model per outcome and the predicted outcome probabilities from each model is stacked to predict any outcome. For the combined models, there is one model for all three outcomes and those probabilities are stacked to predict any outcome. *It should be noted that AKI labels were only available for 4307 of the test patients, and so all AUCs reflect results for only those patients with AKI labels.

In order to better understand the value of modeling outcomes separately, we looked at the correlation between the various outcomes (i.e., the correlation between the prediction of AKI and reintubation, reintubation and mortality, and AKI and mortality). Overall, the various outcomes showed modest correlation with Pearson correlation coefficients ranging from 0.68 to 0.74. These data are shown in Fig. 2.

## Discussion

In this manuscript, we describe the successful creation of model(s) to predict a variety of postoperative outcomes, including AKI, reintubation, mortality, and a combined any postoperative event. These models all performed very well with AUCs ranging from 0.767 to 0.906, and consistently outperformed the ASA physical status score. In efforts to improve our results and, in order to better understand what methodology might improve model performance, we attempted a variety of different techniques, including training a model that had information on all of the outcomes (multitask learning), adding more clinically relevant input features, and feature reduction. None of these modifications significantly improved or reduced DNN model performance. These results are similar to previous work, where we did not see a substantial improvement in performance between LR and DNNs for mortality^24^. In comparing our models to LR and other previously described models (RSI and RQI), we found improvement for AKI but not other outcomes. However, while the AUCs of the various models were similar, we did see some variation in other measures of model performance, such as sensitivity, specificity, precision, and accuracy.

One of the potential advantages of ML is that a single set of features can be used to predict a wide variety of outcomes. In fact, the ability to create models that target specific outcomes is of great potential clinical utility. Differentiating the risk of pulmonary complications as opposed to renal complications can have profound effects on decisions, such as intraoperative fluid management, ventilator settings, and even procedure choice (i.e., use of contrast). Importantly, in looking at the correlations between our predictions, we found only modest correlation. Thus, the risk one complication cannot be used to predict the likelihood of another one.

In an effort to improve overall model performance we attempted a multitask learning technique, as well as adding key features that have been shown in the medical literature to be associated with our outcomes of interest. Despite trying a variety of different feature sets as well as model techniques, we found remarkably consistent AUC results for a given outcome. Even the combined models that suffered from a reduced sample size due to the missing Cr results, had similar AUCs for mortality and reintubation as the individual models for those outcomes. In fact, those models with fewer patients actually had better precision and recall—likely due to the higher incidence of the complications. There are several possible interpretations. One possibility is that our models contained too few features. While 50 or more features are considered robust by traditional statistical standards, ML models often contain hundreds or even millions of features^25^. We attempted to account for this by adding some specific features that are known to be highly associated with our outcomes of interest—features containing data on intraoperative hypotension—with no improvement in results. While this is certainly not conclusive, it does point to a second possible explanation: that there is an upper limit in the predictive ability of any model. To take this concept to its most extreme conclusion, if any model could predict an outcome with 100% certainty it would imply the ability to see the future as there are always some events that happen by chance (i.e., a provider making a syringe swap, or pharmacy releasing the wrong dose of a medication). Without question, some outcomes that are highly multifactorial, or occur further into the future will be harder to predict.

An interesting finding in our results is that while the AUCs of the various models were consistent for a given outcome there was some variation in other measures of model performance, such as sensitivity, specificity, and precision. From our analysis there did not seem to be a clear pattern to these results. Further, even models with similar AUCs sometimes had different overall accuracy (as determined with the McNemar test) for the threshold that optimized the F1 score. We believe that this has two critical implications. First, it highlights the fact that there is no single metric for a “best” model. Rather it is critical that one have specific clinical implementations in mind when designing a model; for example, a model which is to be used a screening test might be optimized for sensitivity while a model used to alter treatment would require a high precision. Models are not “one size fits all”. The other implication of the variability in these performance measures is the need to be fluent in a variety of modeling techniques. If there is indeed no particular pattern which can lead one to determine which techniques will optimize metrics like sensitivity or precision, then creation of models must be undertaken with a clear understanding of their ultimate use. Models which are designed for screening should be created to optimize sensitivity while those that prescribe treatments would be optimized for precision. Developers may be required to try several techniques in an attempt to optimize the actual implementation and the definition of the “best” model will depend on its intended role. Indeed, a key part of this decision may not only be a statistical definition of what is best, but also a consideration for ease of implementation, processing power, model interoperability and other workflow related factors.

In comparing the effectiveness of our models to the other commonly used models (ASA score, RSI, and RQI), we noted that those models preformed well for mortality and reintubation but less well for AKI (and in the case of RSI any outcome). This may be because clinicians, who prescribe the ASA score, generally think about mortality but may be less attuned to other (less correlated outcomes), such as AKI. Further, the RSI and RQI were explicitly created to model mortality as opposed to AKI. Thus, we see that using this model to predict AKI is less effective, a hypothesis supported by the lower correlation between AKI and the mortality model in Fig. 2. This finding supports the need for models that are separately designed to predict different outcomes, as opposed to a “one size fits all” approach.

The biggest limitation to our work is that this is a single-center trial, thus the models that we describe here might not have identical performance at other institutions. ML models often have training sets that number in the hundreds of thousands or millions, in order to capture all possible variabilities and generalize for any population. In order to address this shortcoming, we sought to limit our feature set and using techniques to prevent overfitting. A second limitation of our work is that we lost a large number of cases due to missing preoperative or postoperative creatinine values. This challenge has been faced by others who created models to predict postoperative AKI, such as Kheterpal et al.^28^. This data loss may be one reason why the AUC for the AKI models were lower; however, they still outperformed the ASA score on its own.

Overall, in this manuscript, we were able to create models for a variety of postoperative outcomes using DNNs. We found no one technique to be consistently superior, indicating that those interested in this emerging area should seek to attempt a variety of ML techniques.

## Methods

This manuscript follows the “Guidelines for Developing and Reporting Machine Learning Predictive Models in Biomedical Research: A Multidisciplinary View”^29^. All data used for this study were obtained from this data warehouse and IRB approval (UCLA IRB#15-000518) has been obtained for this retrospective review and waived the requirement for written informed consent.

### EMR data extraction

All data for this study were extracted from the Perioperative Data Warehouse (PDW), a custom-built robust data warehouse containing all patients who have undergone surgery at UCLA, since the implementation of the EMR (EPIC Systems, Madison WI) on March 17th, 2013. The construction of the PDW has been previously described^30^. Briefly, the PDW has a two-stage design. In the first stage, data are extracted from EPIC’s Clarity database into 26 tables organized around three distinct concepts: patients, surgical procedures, and health system encounters. These data are then used to populate a series of 800 distinct measures and metrics, such as procedure duration, readmissions, admission ICD codes, and others.

A list of all surgical cases performed between March 17, 2013 and July 16, 2016 were extracted from the PDW. The UCLA Health System includes two-inpatient medical centers, as well as three ambulatory surgical centers; however, only cases performed in one of the two-inpatient hospitals (including operating room and “off-site” locations) under general anesthesia were included in this analysis. Cases on patients younger than 18 years of age or older than 89 years of age were excluded. In the event that more than one procedure was performed during a given health system encounter only the first case was included.

### Model end point definition

The occurrence of an in-hospital mortality was extracted as a binary event [0, 1] based upon either the presence of a “mortality date” in the EMR between surgery time and discharge, or a discharge disposition of expired combined with a note associated with the death (i.e., death summary and death note). The definition of in-hospital mortality was independent of length of stay in the hospital.

AKI was determined based upon the change from the patient’s baseline Cr_S_ as described in the Acute Kidney Injury Network (AKIN) criteria^31^. Patients were defined as having AKI if they met criteria for any of the AKIN stages based upon changes in their Cr (e.g., had a Cr_S_ >1.5 times their baseline). Patients who lacked either a preoperative or postoperative Cr were excluded only from the AKI and any event models. The preoperative Cr was defined as the most recent Cr within 6 months prior to surgery, and the postoperative Cr was the highest Cr that was obtained between the end of the case and hospital discharge.

Postoperative reintubation was defined as any reintubation prior to hospital discharge and determined using an algorithm that looked for documentation of an endotracheal tube or charting of ventilator settings by a respiratory therapist following surgery. This algorithm has been previously described elsewhere^32^. Briefly, the algorithm uses nursing documentation, airway documentation, and respiratory therapy documentation to triangulate the time of mechanical ventilation after surgery. The algorithm has been shown to outperform manual chart review in a cohort of cardiac surgical patients.

### Data preprocessing

Prior to the model development, missing values were filled with the mean value for the respective feature unless otherwise described in Supplementary Table 1. Details on missing data can be found in Supplementary Table 1. In addition, to account for observations where the value is clinically out of range, values greater than a clinically normal maximum were set to a maximum possible value, as described in previous work^24^. These out of range values were due to the data artifact in the raw EMR data. The data were then randomly divided into training (80%) and test (20%) data sets, with equal % occurrence of each postoperative outcome. Training data were rescaled to have a mean of 0 and standard deviation of 1 per feature. Test data were rescaled with the training data mean and standard deviation.

### Model input features

Each surgical record corresponded to a unique hospital admission and contained 52 features calculated or extracted at the end of surgery (Supplementary Table 2). For the OFS model, these features were selected based upon previous work with a model to predict in-hospital mortality utilizing a subset of 46 features from an original 87 features chosen by clinician consensus (I.S.H., M.C., and E.G.)^24^. The features included descriptive intraoperative vital signs, such as minimum and maximum blood pressure values; summary of drugs and fluids interventions, such as total blood infused, and total vasopressin administered (all features are detailed in Supplementary Table 1). New to this study was the addition of six new features: minutes of case time spent with MAP < 40, 45, 50, 55, 60, and 65 mmHg. These new MAP features were added as potentially relevant features per studies showing the importance of low blood pressure to the risk of AKI and myocardial infarction^33^. For this model, given the addition of six new features, we also chose to remove features with a Pearson’s correlation > 0.9 with other features and were thus left with a reduced feature set (RFS) of 44 features total. Thus, while the overall architecture of this model is similar to aforementioned model to predict mortality, the various models here have somewhat different input features.

### Model development

We utilized five-fold cross validation with the training set (80%) to select for the best performing DNN models’ hyperparameters and architecture. The hyperparameters assessed were number of hidden layers (1–5), number of neurons (10–100), learning rate (0.01, 0.1), and momentum (0.5, 0.9). To avoid overfitting, we also utilized L2 regularization (0.001, 0.0001) and dropout (*p* = 0, 0.5, 0.9; refs. ^34,35^). These hyperparameters and architecture were then used to train a model on the entire training set (80%) prior to testing final model performance on the separate test set (20%). For patients without a preoperative baseline Cr and/or a postoperative Cr, we could not determine postoperative AKI. Those patients were excluded from training for the individual AKI models and the combined models. In total that amounted to exclusion of 38,305 patients or 63.8% of the total sample.

Three separate DNN models were created with each predicting one postoperative outcome of interest: in-hospital mortality, AKI, and reintubation. Specifically, we utilized the same DNN architecture as in our previous work to predict in-hospital mortality, a feedforward network with fully connected layers and a logistic output^24^. A logistic output was chosen so that the output of each outcomes model could be interpreted as probability of each postoperative outcome of interest [0–1]. We utilized stochastic gradient descent with momentum of [0.5, 0.9] and an initial learning rate of [0.01, 0.1], and a batch size of 200. To avoid overfitting, we utilized early stopping with a patience of ten epochs, L2 weight penalty of 0.0001, and dropout with a probability of [0.2, 0.5] (refs. ^28,34,35^). We also assessed DNN architectures of 3–5 hidden layers with [90, 100, 300, 400] neurons per layer, and rectified linear unit and hyperbolic tangent (tanh) activation functions. The loss function was cross entropy. To deal with the highly unbalanced data sets, we also utilized data augmentation during training per our previous work with prediction of in-hospital mortality. Observations positive for reintubation or in-hospital mortality were augmented 100-fold. Observations positive for AKI were augmented threefold. Augmentation was done by adding Gaussian noise taken from a Gaussian distribution with a SD of 0.0001.

To assess if a model could leverage the relationship between the three outcomes (i.e., multitask learning), we also created combined models that output probabilities of all three outcomes at once. The same hyperparameters as the individual models were assessed, with the exception of the use of a batch size of 100.

**Fig. 3:**
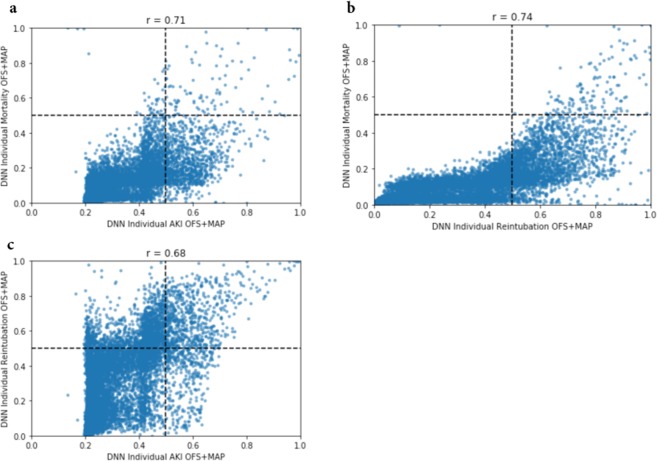
Scatter plot and Pearson correlations for potential outcome pairs. Scatter plot comparison and Pearson correlation (*r*) for predicted probabilities of AKI, mortality, and reintubation from the best performing AUC DNN model with OFS + MAP features. **a** AKI vs Mortality; **b** Reintubation vs Mortality; **c** AKI vs Reintubation.

We were also interested in predicting the probability of the occurrence of any of the three postoperative outcomes. For the combined DNN model, we took the average of the predicted probability outputs for each outcome (Fig. 3). In other words, each predicted probability was given equal weight. The averaged value was considered as the probability of any of the three outcomes occurring. For the individual outcome models (DNN and LR), we took the predicted probability of each respective outcome model per equivalent feature set inputs and averaged the three values (Fig. 3). For example, the outputs of each of the models for AKI, reintubation, and mortality with a RFS were averaged to represent the probability of any outcome occurring.

After choosing the best performing DNN architectures for the RFS, we also assessed the performance of models with two other input feature sets: (1) original 46 features set (OFS) and (2) OFS plus the addition of six new MAP features (OFS + MAP). This was done to assess if the reduction of features improved performance compared to a model with more features, and also to assess if the addition of the clinically significant MAP features not used in previous improved performance overall.

### Model performance

All model performances were assessed on 20% of the data held out from training as a test set. Those patients without an AKI label were excluded from evaluation of test set results for AKI, but not for in-hospital mortality, reintubation, or any outcome results. This is due to the input features of each model independence from the determination of AKI, and so all test patients can have an AKI model predicted probability even if AKI class is unknown. For comparison, we also assessed the performances of the ASA score, RQI (ref. ^36^), RSI (ref. ^27^), and LR models using the same input feature sets as in the DNN. It should be noted that RQI log probability and score were calculated from equations provided in Sigakis et al.^27^. Uncalibrated RSI was calculated using coefficients provided by the original authors and is provided as Supplemental Digital Content in our previous work^24^. A total of 95% confidence intervals for all performance metrics were calculated using bootstrapping with replacement 1000 times from the test set.

Overall model performance was assessed using AUC and average precision (AP) of each model. The precision-recall curve was created by calculating the precision tp/(tp + fp) and recall tp/(tp + fn) at different probability thresholds, where tp, fp, and fn refer to the number of true positives, false positives, and false negatives. The AP score was calculated as the weighted mean of all precisions, with the weight being the increase in recall from the previous threshold^37^.

The *F*1 score, sensitivity, and specificity were calculated for different thresholds for the DNN models. The *F*1 score is a measure of precision and recall, ranging from 0 to 1. It is calculated as $F1 = 2 \times \frac{{precision \;\times\; recall}}{{precision\; + \; recall}}$. For each of the three outcomes, we chose a threshold based on the highest *F*1 score, and assessed the number of true positives, true negatives, false positives, and false negatives, precision, sensitivity, and specificity.

To compare the predictions of the DNN and LR models to each other, we utilized McNemar’s test^38^. McNemar’s test compares the number of correctly predicted samples vs wrongly predicted samples, and where they do and do not predict the same label. If the *p* value of the McNemar test is significant, we can reject the null hypothesis that the two models have the same classification performance. McNemar’s test was performed using the freely available package MLxtend^39^.

All neural network models were developed using Keras. All performance metrics, except for McNemar’s test, and LR models were developed using sci-kit learn^37^.

### Reporting summary

Further information on research design is available in the Nature Research Reporting Summary linked to this article.

## Supplementary information

Supplemental Material

Nature Digital Reporting Summary

## Data Availability

The datasets generated during and/or analyzed during the current study are not publicly available due to institutional restrictions on data sharing and privacy concerns. However, the data are available from the corresponding author on reasonable request.

## Code availability

The code used in this study can be found at https://github.com/cklee219/PostoperativeOutcomes_RiskNet
