# Supplementary material for: Development and validation of a deep neural network model to predict postoperative mortality, acute kidney injury, and reintubation using a single feature set
Source: NPJ Digit Med. 2020 Apr 20;3:58. doi: 10.1038/s41746-020-0248-0 (PMC7170922; doi:10.1038/s41746-020-0248-0)
Supplement: Supplementary file 1 — Supplemental Material [file 41746_2020_248_MOESM1_ESM.pdf]

Feature	Number Patients With Missing Data	Data Fill Type	Mean Value
MAX_ISO	57230	0	1.3
CURRENT_HB	51659	Mean	10.7
MIN_HB	51512	Mean	10.2
MAX_GLUCOSE	51211	Mean	161.0
MIN_GLUCOSE	51211	Mean	121.0
MAX_DES	46961	0	1.0
STARTING_HB	26115	Mean	12.3
MAX_SEVO	20017	0	1.2
BASELINE_GFR	16599	Mean	86.5
MAX_MAP	1510	Mean	114.3
MIN_MAP	1510	Mean	60.2
MIN_MAP_LT_40	535	Mean	0.5
MIN_MAP_LT_45	535	Mean	1.1
MIN_MAP_LT_50	535	Mean	2.6
MIN_MAP_LT_55	535	Mean	6.8
MIN_MAP_LT_60	535	Mean	16.7
MIN_MAP_LT_65	535	Mean	33.6
MAX_PULSE_OX	212	Mean	99.9
MIN_PULSE_OX	212	Mean	91.3
MAX_SBP	207	Mean	164.4
MIN_SBP	207	Mean	79.4
MAX_DBP	207	Mean	94.5
MIN_DBP	207	Mean	43.8
MAX_HR	194	Mean	109.1
MIN_HR	194	Mean	55.7
ASA_SCORE	22	Most common ASA Score 3	2.6

Supplementary Table 1. Summary table of features with missing values in the data set and how missing values were filled.

Feature	Feature Description	In Reduced Feature Set (Y/N)
AGE	Age of the patient in years (note we exclude ages<18 and >89)	Y
ART_LINE_YN	Presence of arterial line	Y
ASA_SCORE	ASA score	Y
BASELINE_GFR	Most recent GFR prior to surgery (only within 365 days)	Y
COLLOID_ML	Total Colloid Transfused	Y
CRYSTALLOID_ML	Total Crystalloid Transfused	Y
CURRENT_HB	Most recent hemoglobin prior to surgery	N
CVC_ANES_YN	Presence of a central venous line	Y
EBL	Total Estimated Blood Loss	Y
EPINEPHRINE_CURRENT_RATE_MCG_KG_MIN	End of case rate of Epinephrine	Y
EPINEPHRINE_MAX_RATE_MCG_KG_MIN	Highest infusion rate of epinephrine during the case	Y
ESMOLOL_CURRENT_RATE_MCG_KG_MIN	End of case Rate of Esmolol	Y
ESMOLOL_MAX_RATE_MCG_KG_MIN	Highest infusion rate of esmolol during the case	Y
MAX_DBP	Maximum Diastolic BP for the case	Y
MAX_DES	Maximum MAC of desflurane during the case (note this is not age adjusted)	Y
MAX_GLUCOSE	Maximum Glucose for the Case	Y
MAX_HR	Maximum Heart Rate for the case	Y
MAX_ISO	Maximum MAC of isoflurane during the case (note this is not age adjusted)	Y
MAX_MAP	Maximum Mean Arterial Pressue for the case	Y
MAX_PULSE_OX	Maximum Pluse OX for the case	Y
MAX_SBP	Maximum Systolic BP for the case	Y
MAX_SEVO	Maximum MAC of sevoflurane during the case (note this is not age adjusted)	Y
MILRINONE_CURRENT_RATE_MCG_KG_MIN	End of case infusion rate of milrinone	N
MILRINONE_MAX_RATE_MCG_KG_MIN	Highest infusion rate of milrone during the case	Y
MIN_DBP	Minimum Diastolic BP for the case	Y
MIN_GLUCOSE	Minimum Glucose for the Case	Y
MIN_HB	Minimum Hemoglobin during the case	Y
MIN_HR	Minimum Heart Rate for the case	Y
MIN_MAP	Minimum Mean Arterial Pressue for the case	Y
MIN_MAP_LT_40*	Minutes MAP < 40 mmHg	Y
MIN_MAP_LT_45*	Minutes MAP < 45 mmHg	N
MIN_MAP_LT_50*	Minutes MAP < 50 mmHg	N
MIN_MAP_LT_55*	Minutes MAP < 55 mmHg	N
MIN_MAP_LT_60*	Minutes MAP < 60 mmHg	N
MIN_MAP_LT_65*	Minutes MAP < 65 mmHg	Y
MIN_PULSE_OX	Minimum Pluse OX for the case	Y
MIN_SBP	Minimum Systolic BP for the case	Y
NICARDIPINE_CURRENT_RATE_MG_HR	End of case Rate of Nicardipine	Y
NICARDIPINE_MAX_RATE_MG_HR	Highest infusion rate of nicardipine during the case	Y
NITRIC_OXIDE_YN	Nitric Oxide Used for the Case	Y
NITROGLYCERIN_CURRENT_RATE_MCG_MIN	End of case Rate of Nitroglycerin	Y
NITROGLYCERIN_MAX_RATE_MCG_MIN	Highest infusion rate of nitroglycerin during the case	Y
NITROPRUSSIDE_CURRENT_RATE_MCG_KG_MIN	End of case Rate of Nitroprusside	Y
NITROPRUSSIDE_MAX_RATE_MCG_KG_MIN	Highest infusion rate of nitroprusside during the case	N
PA_LINE_YN	Presence of pulmonary artery catheter	Y

PHENYLEPHRINE_CURRENT_RATE_MCG_MIN	End of case Rate of Phenylephrine	Y
PHENYLEPHRINE_MAX_RATE_MCG_MIN	Highest infusion rate of phenylephrine during the case	Y
STARTING_HB	Starting Hemoglobin	Y
UOP	Total Urine Output	Y
VASO_CURRENT_RATE_UNITS_HR	End of case Rate of Vasopressin	Y
VASOPRESSIN_MAX_RATE_UNITS_HR	Highest infusion rate of vasopressin during the case	N
XFUSION_RBC_ML	Total Red Blood Cells Transfused	Y

Supplementary Table 2. Description of model input features. There are 52 features total, of which 6 are new MAP features* and 44 are included in the reduced feature set.
*Note new MAP features not utilized in previous work

AKI*

Score	Threshold	F1 Score (95% CI)	Sensitivity (95% CI)	Specificity (95% CI)	Precision (95% CI)	TN	FP	FN	TP
ASA	3	0.412 (0.393 - 0.43)	0.914 (0.896 - 0.93)	0.27 (0.255 - 0.284)	0.266 (0.251 - 0.281)	901	2439	83	884
LR OFS	0.100071	0.423 (0.404 - 0.442)	0.902 (0.881 - 0.92)	0.315 (0.299 - 0.331)	0.276 (0.261 - 0.292)	1052	2288	95	872
LR OFS + MAP features	0.10074	0.424 (0.405 - 0.444)	0.901 (0.88 - 0.92)	0.321 (0.306 - 0.338)	0.278 (0.262 - 0.294)	1073	2267	96	871
LR RFS	0.101606	0.426 (0.407 - 0.445)	0.902 (0.881 - 0.92)	0.325 (0.309 - 0.341)	0.279 (0.263 - 0.294)	1084	2256	95	872
DNN Individual OFS	0.339436	0.44 (0.42 - 0.458)	0.904 (0.883 - 0.92)	0.36 (0.346 - 0.376)	0.29 (0.274 - 0.306)	1204	2136	93	874
DNN Individual OFS + MAP features	0.258765	0.452 (0.432 - 0.469)	0.901 (0.881 - 0.917)	0.396 (0.38 - 0.412)	0.302 (0.285 - 0.318)	1323	2017	96	871
DNN Individual RFS	0.333397	0.448 (0.428 - 0.466)	0.905 (0.884 - 0.921)	0.381 (0.366 - 0.398)	0.297 (0.281 - 0.313)	1273	2067	92	875
DNN Combined OFS	0.681036	0.452 (0.432 - 0.471)	0.902 (0.881 - 0.92)	0.395 (0.379 - 0.412)	0.301 (0.285 - 0.318)	1319	2021	95	872
DNN Combined OFS + MAP features	0.694522	0.447 (0.427 - 0.465)	0.901 (0.881 - 0.918)	0.383 (0.367 - 0.4)	0.297 (0.281 - 0.313)	1279	2061	96	871
DNN Combined RFS	0.690984	0.448 (0.428 - 0.467)	0.902 (0.883 - 0.919)	0.386 (0.37 - 0.403)	0.298 (0.282 - 0.315)	1288	2052	95	872

Reintubation

Score	Threshold	F1 Score (95% CI)	Sensitivity (95% CI)	Specificity (95% CI)	Precision (95% CI)	TN	FP	FN	TP
ASA	3	0.043 (0.037 - 0.051)	0.943 (0.905 - 0.976)	0.442 (0.434 - 0.451)	0.022 (0.019 - 0.026)	5237	6600	9	150
LR OFS	0.004	0.043 (0.036 - 0.05)	0.918 (0.873 - 0.96)	0.453 (0.444 - 0.461)	0.022 (0.019 - 0.026)	5358	6479	13	146
LR OFS + MAP features	0.004	0.043 (0.036 - 0.05)	0.918 (0.873 - 0.96)	0.454 (0.446 - 0.463)	0.022 (0.019 - 0.026)	5377	6460	13	146
LR RFS	0.005193	0.05 (0.043 - 0.059)	0.906 (0.855 - 0.952)	0.543 (0.535 - 0.552)	0.026 (0.022 - 0.03)	6430	5407	15	144
DNN Individual OFS	0.373748	0.065 (0.056 - 0.076)	0.906 (0.857 - 0.949)	0.654 (0.646 - 0.662)	0.034 (0.029 - 0.039)	7738	4099	15	144
DNN Individual OFS + MAP features	0.385977	0.065 (0.055 - 0.076)	0.906 (0.856 - 0.951)	0.652 (0.643 - 0.66)	0.034 (0.028 - 0.04)	7716	4121	15	144
DNN Individual RFS	0.346943	0.065 (0.055 - 0.075)	0.906 (0.856 - 0.951)	0.65 (0.642 - 0.658)	0.034 (0.028 - 0.039)	7691	4146	15	144
DNN Combined OFS	0.341994	0.055 (0.047 - 0.064)	0.906 (0.861 - 0.949)	0.583 (0.575 - 0.591)	0.028 (0.024 - 0.033)	6901	4936	15	144
DNN Combined OFS + MAP features	0.347518	0.046 (0.039 - 0.054)	0.906 (0.857 - 0.95)	0.497 (0.488 - 0.506)	0.024 (0.02 - 0.028)	5884	5953	15	144
DNN Combined RFS	0.378933	0.055 (0.046 - 0.063)	0.906 (0.859 - 0.951)	0.58 (0.572 - 0.589)	0.028 (0.024 - 0.033)	6864	4973	15	144

Mortality

Score	Threshold	F1 Score (95% CI)	Sensitivity (95% CI)	Specificity (95% CI)	Precision (95% CI)	TN	FP	FN	TP
ASA	3	0.025 (0.02 - 0.03)	0.977 (0.938 - 1.0)	0.44 (0.432 - 0.449)	0.013 (0.01 - 0.015)	5244	6665	2	85
LR OFS	0.004	0.04 (0.033 - 0.049)	0.92 (0.853 - 0.975)	0.682 (0.673 - 0.69)	0.021 (0.017 - 0.025)	8117	3792	7	80
LR OFS + MAP features	0.004	0.041 (0.033 - 0.049)	0.92 (0.853 - 0.975)	0.682 (0.674 - 0.691)	0.021 (0.017 - 0.025)	8127	3782	7	80
LR RFS	0.005	0.049 (0.04 - 0.06)	0.908 (0.841 - 0.964)	0.744 (0.737 - 0.752)	0.025 (0.02 - 0.031)	8861	3048	8	79
DNN Individual OFS	0.15	0.042 (0.033 - 0.051)	0.908 (0.841 - 0.963)	0.697 (0.689 - 0.705)	0.021 (0.017 - 0.026)	8298	3611	8	79
DNN Individual OFS + MAP features	0.127	0.044 (0.035 - 0.053)	0.908 (0.835 - 0.966)	0.709 (0.701 - 0.718)	0.022 (0.018 - 0.027)	8449	3460	8	79
DNN Individual RFS	0.175	0.042 (0.034 - 0.051)	0.908 (0.843 - 0.965)	0.698 (0.69 - 0.707)	0.022 (0.017 - 0.026)	8316	3593	8	79
DNN Combined OFS	0.309117	0.043 (0.034 - 0.053)	0.908 (0.845 - 0.966)	0.706 (0.698 - 0.713)	0.022 (0.017 - 0.027)	8408	3501	8	79
DNN Combined OFS + MAP features	0.367254	0.044 (0.035 - 0.054)	0.908 (0.841 - 0.966)	0.712 (0.703 - 0.719)	0.022 (0.018 - 0.028)	8474	3435	8	79
DNN Combined RFS	0.427749	0.055 (0.044 - 0.067)	0.908 (0.843 - 0.965)	0.772 (0.765 - 0.779)	0.028 (0.022 - 0.035)	9194	2715	8	79

Any Outcome

Score	Threshold	F1 Score (95% CI)	Sensitivity (95% CI)	Specificity (95% CI)	Precision (95% CI)	TN	FP	FN	TP
ASA	3	0.25 (0.238 - 0.263)	0.917 (0.901 - 0.933)	0.472 (0.462 - 0.481)	0.145 (0.137 - 0.153)	5157	5772	89	978
LR OFS	0.038592	0.26 (0.247 - 0.274)	0.901 (0.882 - 0.919)	0.51 (0.5 - 0.52)	0.152 (0.143 - 0.161)	5572	5357	106	961
LR OFS + MAP features	0.03759	0.256 (0.243 - 0.269)	0.905 (0.888 - 0.923)	0.495 (0.486 - 0.505)	0.149 (0.14 - 0.158)	5411	5518	101	966
LR RFS	0.037499	0.255 (0.243 - 0.268)	0.904 (0.887 - 0.923)	0.494 (0.484 - 0.504)	0.149 (0.14 - 0.157)	5397	5532	102	965
DNN Individual OFS	0.247454	0.305 (0.29 - 0.32)	0.901 (0.882 - 0.918)	0.61 (0.601 - 0.619)	0.184 (0.173 - 0.194)	6664	4265	106	961
DNN Individual OFS + MAP features	0.248621	0.308 (0.293 - 0.322)	0.903 (0.884 - 0.92)	0.613 (0.604 - 0.622)	0.186 (0.175 - 0.196)	6697	4232	103	964
DNN Individual RFS	0.239795	0.327 (0.311 - 0.342)	0.902 (0.883 - 0.919)	0.648 (0.639 - 0.656)	0.2 (0.188 - 0.211)	7078	3851	105	962
DNN Combined OFS	0.411049	0.304 (0.289 - 0.319)	0.901 (0.882 - 0.918)	0.608 (0.599 - 0.617)	0.183 (0.172 - 0.194)	6640	4289	106	961
DNN Combined OFS + MAP features	0.441431	0.296 (0.28 - 0.31)	0.902 (0.883 - 0.919)	0.591 (0.581 - 0.6)	0.177 (0.166 - 0.187)	6456	4473	105	962
DNN Combined RFS	0.445316	0.292 (0.276 - 0.306)	0.901 (0.882 - 0.919)	0.583 (0.574 - 0.593)	0.174 (0.164 - 0.184)	6375	4554	106	961

Supplementary Table 3a. **Best threshold chosen by highest sensitivity > 0.90**. Comparison of F1 score, sensitivity and specificity with best thresholds for acute kidney injury (AKI), reintubation, mortality, and any outcome with 95% CIs for the Test Set (N=11,996) for the ASA score, logistic regression (LR) models, deep neural networks predicting individual outcomes (DNN individual), and deep neural networks predicting all 3 outcomes (DNN combined). Each model was also evaluated for each feature set combination of original feature set (OFS), OFS + the minimum MAP features (OFS + MAP), and reduced feature set (RFS). Note that for the LR and individual models, there is one model per outcome and the predicted outcome probabilities from each model is stacked to predict any outcome. For the combined models, there is one model for all 3 outcomes and those probabilities are stacked to predict any outcome. *It should be noted that AKI labels were only available for 4307 of the test patients, and so all results for AKI are from those patients with AKI labels.

**AKI***									
Score	thresh	f1	sens	spec	prec	tn	fp	fn	tp
ASA	5	0.022 (0.01 - 0.035)	0.011 (0.005 - 0.018)	0.997 (0.995 - 0.998)	0.5 (0.278 - 0.7)	3329	11	95611	
LR OFS	0.379071	0.51 (0.481 - 0.539)	0.459 (0.427 - 0.489)	0.901 (0.891 - 0.911)	0.573 (0.538 - 0.608)	3009	331	523444	
LR RFS	0.380606	0.512 (0.483 - 0.54)	0.463 (0.431 - 0.494)	0.9 (0.89 - 0.91)	0.573 (0.539 - 0.609)	3006	334	519448	
LR OFS + MAP Features	0.38074	0.513 (0.485 - 0.542)	0.463 (0.432 - 0.494)	0.901 (0.891 - 0.911)	0.575 (0.542 - 0.61)	3009	331	519448	
DNN Combined RFS	0.892984	0.525 (0.497 - 0.553)	0.478 (0.447 - 0.508)	0.901 (0.891 - 0.91)	0.583 (0.549 - 0.618)	3009	331	505462	
DNN Combined OFS	0.923036	0.539 (0.512 - 0.568)	0.494 (0.463 - 0.525)	0.901 (0.891 - 0.911)	0.592 (0.56 - 0.626)	3011	329	489478	
DNN Combined OFS + MAP Features	0.929522	0.515 (0.487 - 0.546)	0.466 (0.434 - 0.497)	0.901 (0.89 - 0.911)	0.576 (0.541 - 0.613)	3008	332	516451	
DNN Individual OFS	0.468436	0.512 (0.483 - 0.541)	0.462 (0.432 - 0.495)	0.9 (0.89 - 0.91)	0.573 (0.538 - 0.608)	3007	333	520447	
DNN Individual RFS	0.452397	0.518 (0.49 - 0.546)	0.468 (0.439 - 0.5)	0.901 (0.89 - 0.911)	0.579 (0.544 - 0.612)	3010	330	514453	
DNN Individual OFS + MAP Features	0.495765	0.537 (0.51 - 0.564)	0.493 (0.463 - 0.524)	0.9 (0.89 - 0.91)	0.589 (0.557 - 0.624)	3007	333	490477	
**Reintubation**									
Score	thresh	f1	sens	spec	prec	tn	fp	fn	tp
ASA	4	0.152 (0.121 - 0.182)	0.44 (0.361 - 0.517)	0.941 (0.937 - 0.945)	0.092 (0.072 - 0.112)	11142695		89	70
LR OFS	0.023	0.148 (0.123 - 0.173)	0.654 (0.581 - 0.73)	0.903 (0.898 - 0.908)	0.083 (0.068 - 0.099)	10691114655			104
LR RFS	0.023193	0.147 (0.121 - 0.173)	0.648 (0.572 - 0.724)	0.904 (0.898 - 0.909)	0.083 (0.067 - 0.098)	10695114256			103
LR OFS + MAP Features	0.023	0.144 (0.119 - 0.169)	0.635 (0.562 - 0.709)	0.904 (0.898 - 0.909)	0.082 (0.066 - 0.097)	10699113858			101
DNN Combined RFS	0.567933	0.126 (0.102 - 0.15)	0.566 (0.49 - 0.641)	0.9 (0.895 - 0.906)	0.071 (0.057 - 0.086)	10657118069			90
DNN Combined OFS	0.619994	0.13 (0.107 - 0.154)	0.585 (0.515 - 0.667)	0.9 (0.895 - 0.905)	0.073 (0.059 - 0.088)	10655118266			93
DNN Combined OFS + MAP Features	0.613518	0.127 (0.105 - 0.151)	0.572 (0.497 - 0.649)	0.9 (0.895 - 0.906)	0.071 (0.058 - 0.086)	10655118268			91
DNN Individual OFS	0.551748	0.146 (0.121 - 0.171)	0.66 (0.58 - 0.73)	0.9 (0.895 - 0.906)	0.082 (0.067 - 0.098)	10659117854			105
DNN Individual RFS	0.532943	0.152 (0.126 - 0.177)	0.692 (0.611 - 0.765)	0.901 (0.895 - 0.906)	0.085 (0.07 - 0.101)	10660117749			110
DNN Individual OFS + MAP Features	0.574977	0.147 (0.122 - 0.173)	0.667 (0.585 - 0.736)	0.9 (0.895 - 0.906)	0.083 (0.068 - 0.098)	10659117853			106
**Mortality**									
Score	thresh	f1	sens	spec	prec	tn	fp	fn	tp
ASA	4	0.113 (0.086 - 0.143)	0.552 (0.451 - 0.654)	0.94 (0.936 - 0.944)	0.063 (0.047 - 0.081)	11192717		39	48
LR OFS	0.013								

Score	thresh	f1	sens	spec	prec	tn	fp	fn	tp
ASA	4	0.36 (0.335 - 0.387)	0.309 (0.283 - 0.337)	0.96 (0.957 - 0.964)	0.431 (0.399 - 0.468)	10494	435	737	330
LR OFS	0.094592	0.485 (0.461 - 0.508)	0.648 (0.619 - 0.678)	0.9 (0.895 - 0.906)	0.388 (0.365 - 0.41)	9837	1092	376	691
LR RFS	0.095499	0.485 (0.462 - 0.507)	0.648 (0.619 - 0.679)	0.9 (0.895 - 0.906)	0.388 (0.365 - 0.41)	9838	1091	376	691
LR OFS + MAP Features	0.09559	0.483 (0.46 - 0.506)	0.642 (0.614 - 0.674)	0.901 (0.895 - 0.906)	0.387 (0.365 - 0.41)	9846	1083	382	685
DNN Individual OFS	0.376454	0.468 (0.445 - 0.49)	0.613 (0.585 - 0.643)	0.902 (0.896 - 0.907)	0.378 (0.356 - 0.4)	9853	1076	413	654
DNN Individual RFS	0.376621	0.462 (0.439 - 0.484)	0.605 (0.577 - 0.636)	0.901 (0.896 - 0.906)	0.373 (0.351 - 0.395)	9844	1085	421	646
DNN Individual OFS + MAP Features	0.380795	0.473 (0.45 - 0.496)	0.624 (0.596 - 0.655)	0.901 (0.895 - 0.906)	0.38 (0.358 - 0.402)	9843	1086	401	666
DNN Combined OFS	0.657049	0.469 (0.447 - 0.491)	0.617 (0.589 - 0.647)	0.901 (0.896 - 0.906)	0.378 (0.357 - 0.401)	9846	1083	409	658
DNN Combined OFS + MAP Features	0.658431	0.468 (0.445 - 0.491)	0.619 (0.59 - 0.648)	0.9 (0.895 - 0.905)	0.377 (0.353 - 0.4)	9837	1092	407	660
DNN Combined RFS	0.627316	0.443 (0.42 - 0.466)	0.575 (0.543 - 0.605)	0.9 (0.895 - 0.906)	0.36 (0.338 - 0.383)	9839	1090	454	613

Supplementary Table 3b. **Best threshold chosen by highest specificity closest to 0.90**. Comparison of F1 score, sensitivity and specificity with best thresholds for acute kidney injury (AKI), reintubation, mortality, and any outcome with 95% CIs for the Test Set (N=11,996) for the ASA score, logistic regression (LR) models, deep neural networks predicting individual outcomes (DNN individual), and deep neural networks predicting all 3 outcomes (DNN combined). Each model was also evaluated for each feature set combination of original feature set (OFS), OFS + the minimum MAP features (OFS + MAP), and reduced feature set (RFS). Note that for the LR and individual models, there is one model per outcome and the predicted outcome probabilities from each model is stacked to predict any outcome. For the combined models, there is one model for all 3 outcomes and those probabilities are stacked to predict any outcome. *It should be noted that AKI labels were only available for 4307 of the test patients, and so all results for AKI are from those patients with AKI labels.

**AKI***									
model	thresh	f1	sens	spec	prec	tn	fp	fn	tp
ASA	5	0.022 (0.01 - 0.035)	0.011 (0.005 - 0.018)	0.997 (0.995 - 0.998)	0.5 (0.278 - 0.7)	3329	11	956	11
LR OFS	0.980071	0.022 (0.01 - 0.037)	0.011 (0.005 - 0.019)	1.0 (0.999 - 1.0)	0.917 (0.727 - 1.0)	3339	1	956	11
LR RFS	0.980606	0.024 (0.01 - 0.039)	0.012 (0.005 - 0.02)	1.0 (0.999 - 1.0)	0.923 (0.737 - 1.0)	3339	1	955	12
LR OFS + MAP Features	0.97974	0.022 (0.01 - 0.037)	0.011 (0.005 - 0.019)	1.0 (0.999 - 1.0)	0.917 (0.727 - 1.0)	3339	1	956	11
DNN Combined RFS	0.986984	0.06 (0.04 - 0.08)	0.031 (0.02 - 0.042)	0.999 (0.998 - 1.0)	0.882 (0.75 - 0.971)	3336	4	937	30
DNN Combined OFS	0.982036	0.188 (0.158 - 0.22)	0.105 (0.087 - 0.125)	0.995 (0.992 - 0.997)	0.857 (0.786 - 0.915)	3323	17	865	102
DNN Combined OFS + MAP Features	0.994522	0.084 (0.063 - 0.109)	0.044 (0.033 - 0.058)	0.997 (0.995 - 0.999)	0.827 (0.719 - 0.922)	3331	9	924	43
DNN Individual OFS	0.954436	0.002 (0.0 - 0.006)	0.001 (0.0 - 0.003)	1.0 (1.0 - 1.0)	1.0 (1.0 - nan)	3340	0	966	1
DNN Individual RFS	0.979397	0.002 (0.0 - 0.006)	0.001 (0.0 - 0.003)	1.0 (1.0 - 1.0)	1.0 (1.0 - nan)	3340	0	966	1
DNN Individual OFS + MAP Features	0.698765	0.137 (0.111 - 0.164)	0.074 (0.06 - 0.09)	0.997 (0.995 - 0.999)	0.889 (0.814 - 0.952)	3331	9	895	72
**Reintubation**									
model	thresh	f1	sens	spec	prec	tn	fp	fn	tp
ASA	5	0.053 (0.011 - 0.105)	0.031 (0.007 - 0.064)	0.998 (0.997 - 0.999)	0.167 (0.042 - 0.32)	11812	25	154	5
LR OFS	0.472	0.012 (0.0 - 0.039)	0.006 (0.0 - 0.02)	1.0 (1.0 - 1.0)	0.333 (0.0 - nan)	11835	2	158	1
LR RFS	0.391193	0.036 (0.0 - 0.079)	0.019 (0.0 - 0.042)	1.0 (0.999 - 1.0)	0.5 (0.0 - 1.0)	11834	3	156	3
LR OFS + MAP Features	0.454	0.025 (0.0 - 0.06)	0.013 (0.0 - 0.031)	1.0 (1.0 - 1.0)	0.667 (0.0 - nan)	11836	1	157	2
DNN Combined RFS	0.950933	0.035 (0.0 - 0.078)	0.019 (0.0 - 0.042)	0.999 (0.999 - 1.0)	0.273 (0.0 - 0.571)	11829	8	156	3
DNN Combined OFS	0.948994	0.064 (0.023 - 0.112)	0.038 (0.013 - 0.069)	0.998 (0.997 - 0.999)	0.214 (0.077 - 0.364)	11815	22	153	6
DNN Combined OFS + MAP Features	0.970518	0.034 (0.0 - 0.07)	0.019 (0.0 - 0.04)	0.999 (0.998 - 0.999)	0.188 (0.0 - 0.385)	11824	13	156	3
DNN Individual OFS	0.984748	0.024 (0.0 - 0.059)	0.013 (0.0 - 0.032)		0.25 (0.0 - 0.6)	11831	6	157	2

DNN Individual RFS	1	0.023 (0.0 - 0.07)	0.011 (0.0 - 0.036)	1.0 (1.0 - 1.0)	1.0 (1.0 - nan)	119090		86	1
DNN Individual OFS + MAP Features	1	0.023 (0.0 - 0.07)	0.011 (0.0 - 0.036)	1.0 (1.0 - 1.0)	1.0 (1.0 - nan)	119090		86	1
**Any Outcome**									
model	thresh	f1	sens	spec	prec	tn	fp	fn	tp
ASA	5	0.04 (0.024 - 0.059)	0.021 (0.012 - 0.031)0.999 (0.999 - 1.0)		0.733 (0.576 - 0.893)109218			104522	
LR OFS	0.6955920.007 (0.002 - 0.015)0.004 (0.001 - 0.008)1.0 (1.0 - 1.0)				1.0 (1.0 - 1.0)	109290		10634	
LR RFS	0.6954990.004 (0.0 - 0.01)		0.002 (0.0 - 0.005)	1.0 (1.0 - 1.0)	1.0 (1.0 - nan)	109290		10652	
LR OFS + MAP Features	0.69359	0.007 (0.002 - 0.015)0.004 (0.001 - 0.008)1.0 (1.0 - 1.0)			1.0 (1.0 - 1.0)	109290		10634	
DNN Individual OFS	0.8334540.019 (0.008 - 0.031)0.009 (0.004 - 0.016)1.0 (1.0 - 1.0)				1.0 (1.0 - 1.0)	109290		105710	
DNN Individual RFS	0.9146210.015 (0.006 - 0.025)0.007 (0.003 - 0.013)1.0 (1.0 - 1.0)				1.0 (1.0 - 1.0)	109290		10598	
DNN Individual OFS + MAP Features	0.9467950.004 (0.0 - 0.009)		0.002 (0.0 - 0.005)	1.0 (1.0 - 1.0)	1.0 (1.0 - nan)	109290		10652	
DNN Combined OFS	0.9640490.054 (0.037 - 0.073)0.028 (0.019 - 0.038)1.0 (0.999 - 1.0)				0.882 (0.767 - 0.972)109254			103730	
DNN Combined OFS + MAP Features0.9754310.046 (0.031 - 0.063)0.023 (0.016 - 0.032)1.0 (1.0 - 1.0)					0.926 (0.818 - 1.0)	109272		104225	
DNN Combined RFS	0.9583160.026 (0.013 - 0.039)0.013 (0.007 - 0.02)			1.0 (1.0 - 1.0)	0.875 (0.7 - 1.0)	109272		105314	

Supplementary Table 3c. **Best threshold chosen by highest Precision**. Comparison of F1 score, sensitivity and specificity with best thresholds for acute kidney injury (AKI), reintubation, mortality, and any outcome with 95% CIs for the Test Set (N=11,996) for the ASA score, logistic regression (LR) models, deep neural networks predicting individual outcomes (DNN individual), and deep neural networks predicting all 3 outcomes (DNN combined). Each model was also evaluated for each feature set combination of original feature set (OFS), OFS + the minimum MAP features (OFS + MAP), and reduced feature set (RFS). Note that for the LR and individual models, there is one model per outcome and the predicted outcome probabilities from each model is stacked to predict any outcome. For the combined models, there is one model for all 3 outcomes and those probabilities are stacked to predict any outcome. *It should be noted that AKI labels were only available for 4307 of the test patients, and so all results for AKI are from those patients with AKI labels.
